# Hypertension management in rural primary care facilities in Zambia: a mixed methods study

**DOI:** 10.1186/s12913-017-2063-0

**Published:** 2017-02-03

**Authors:** Lily D. Yan, Cindy Chirwa, Benjamin H. Chi, Samuel Bosomprah, Ntazana Sindano, Moses Mwanza, Dennis Musatwe, Mary Mulenga, Roma Chilengi

**Affiliations:** 1 Primary Care and Health Systems Department, Center for Infectious Disease Research Zambia, Lusaka, Zambia; 2 Stanford University School of Medicine, Stanford, CA USA; 3 Department of Obstetrics and Gynecology, University of North Carolina School of Medicine, Chapel Hill, NC USA; 4 Department of Biostatistics, School of Public Health, University of Ghana, Accra, Ghana

**Keywords:** Hypertension, Zambia, Mixed methods, Antihypertensive medication, Performance indicators, Quality improvement

## Abstract

**Background:**

Improved primary health care is needed in developing countries to effectively manage the growing burden of hypertension. Our objective was to evaluate hypertension management in Zambian rural primary care clinics using process and outcome indicators to assess the screening, monitoring, treatment and control of high blood pressure.

**Methods:**

Better Health Outcomes through Mentoring and Assessment (BHOMA) is a 5-year, randomized stepped-wedge trial of improved clinical service delivery underway in 46 rural Zambian clinics. Clinical data were collected as part of routine patient care from an electronic medical record system, and reviewed for site performance over time according to hypertension related indicators: screening (blood pressure measurement), management (recorded diagnosis, physical exam or urinalysis), treatment (on medication), and control. Quantitative data was used to develop guides for qualitative in-depth interviews, conducted with health care providers at a proportional sample of half (20) of clinics. Qualitative data was iteratively analyzed for thematic content.

**Results:**

From January 2011 to December 2014, 318,380 visits to 46 primary care clinics by adults aged ≥ 25 years with blood pressure measurements were included. Blood pressure measurement at vital sign screening was initially high at 89.1% overall (range: 70.1–100%), but decreased to 62.1% (range: 0–100%) by 48 months after intervention start. The majority of hypertensive patients made only one visit to the clinics (57.8%). Out of 9022 patients with at least two visits with an elevated blood pressure, only 49.3% had a chart recorded hypertension diagnosis. Process indicators for monitoring hypertension were <10% and did not improve with time. In in-depth interviews, antihypertensive medication shortages were common, with 15/20 clinics reporting hydrochlorothiazide-amiloride stockouts. Principal challenges in hypertension management included 1) equipment and personnel shortages, 2) provider belief that multiple visits were needed before official management, 3) medication stock-outs, leading to improper prescriptions and 4) poor patient visit attendance.

**Conclusions:**

Our findings suggest that numerous barriers stand in the way of hypertension diagnosis and management in Zambian primary health facilities. Future work should focus on performance indicator development and validation in low resource contexts, to facilitate regular and systematic data review to improve patient outcomes.

**Trial registration:**

ClinicalTrials.gov, Identifier NCT01942278. Date of Registration: September 2013.

**Electronic supplementary material:**

The online version of this article (doi:10.1186/s12913-017-2063-0) contains supplementary material, which is available to authorized users.

## Background

Globally, noncommunicable diseases (NCD) are the leading cause of death, resulting in greater mortality than every other cause combined. Almost 80% of NCD deaths occur in low and middle income countries (LMIC), with cardiovascular diseases (CVD) claiming the greatest percentage [1, 2]. In Zambia, NCDs accounted for 23% of total deaths in 2008, and is expected to grow rapidly in the coming decades as the country continues the epidemiological transition [2]. There is increasing evidence that a robust primary care system can not only handle the growing NCD burden, but also manage risk factors like hypertension for CVD, to prevent disease progression [3, 4].

In this context, performance monitoring is critical to develop, support and sustain effective delivery of proven therapies in primary care for NCDs. Quality improvement provides an iterative framework to monitor multilevel contributors to patient outcomes by using routine health program data to examine what system elements need to be changed [5, 6]. Measurable performance markers are broken down into indicators examining structure (physical facility), process (diagnosis and treatment) and outcome (mortality, recovery) indicators [7, 8]. When linked with pay for performance, indicators accelerate improvements in quality in both developed and developing settings [9, 10].

Existing performance indicators for hypertension were developed in North America and Europe through a modified Delphi process at the national level, with expert panel review of the scientific literature and evaluation for feasibility [11–14]. In the United States, assessments based on 28 process indicators for hypertension showed that only 57% of patients received optimal care, and higher quality care as evidenced by the indicators was associated with a higher likelihood of blood pressure control [11]. Unfortunately, no formal process for creating and validating performance indicators currently exists in Zambia, although there is growing interest in quality improvement [15, 16]. Indicators from the United States or the United Kingdom are not directly applicable to developing countries, where feasibility of indicator measurement may be drastically different. There exists a need to develop locally appropriate quality improvement indicators.

This paper presents data on novel retrospectively generated process and outcome indicators for hypertension management, informed by those from Western countries, but adapted to the Zambian primary care clinics enrolled in the Better Health outcomes through Mentoring and Assessment (BHOMA) study.

## Methods

### Study setting and design

Better Health Outcomes through Mentoring and Assessment (BHOMA) is a 5-year, randomized stepped-wedge trial of improved clinical service delivery underway in 46 rural government clinics in Chongwe, Kafue and Luangwa districts of Zambia, which represent almost all publically run rural clinics in Lusaka province. As such, they capture the full range of size, patient population and services available in rural Zambian government primary care clinics. Many rural clinics are staffed by a single nurse or clinical officer, and have frequent equipment or medication shortages. BHOMA aims to improve primary healthcare through use of standardized protocols for common visits, onsite electronic medical records (EMR), and ongoing mentoring to improve key indicators [17]. BHOMA began with pilot sites in Aug 2010, with the EMR operational starting Jan 2011; thus all data from Jan 2011 to Dec 2014 are included here.

District based Quality Improvement teams of 5–8 specially trained nurses and clinical officers make regular visits at participating clinics for mentoring meetings, record review and general support. Community volunteers were also trained to perform non-specialist tasks as clinic support workers, including patient file management and vital signs. We used an explanatory sequential design by conducting a quantitative analysis, which was then explained through a qualitative follow up component.

### Study subjects

For the quantitative component, all visits by patients aged ≥25 years across all 46 health facilities were included. For the qualitative part, due to logistical constraints a proportional sample of 20 out of 46 clinics supported through BHOMA were chosen for facility audits (10 in Chongwe, 7 in Kafue, 3 in Luangwa). Clinics were stratified by district, and ranked according to percent of total visits with a missing blood pressure (BP) measurement. The top and bottom quarter were chosen to represent the best performing and worst performing clinics. At each clinic, one healthcare provider who staffed the outpatient department was chosen for an in-depth interview.

### Data collection

Routine clinical data on patient demographics, BP measurements, diagnosis, physical exam, lab tests, medication prescription and visits dates were extracted from the EMR. For the qualitative component, facility audits focusing on available equipment, medication stocks and staffing were conducted as part of routine visits by trained members of the BHOMA Quality Improvement teams at half of the clinics, once during March-April 2015. Semi-structured in-depth interviews with health care providers and a representative from the central medication distribution agency in Zambia, Medical Stores Limited, were also completed during these routine visits. Interviews were conducted by bilingual study staff in English or a local language (Nyanja), depending on the preference of the interviewee. Interviews were recorded, transcribed and translated to English if necessary.

### Data analysis

Based on the available literature and data [11–13, 18], we generated process and outcome indicators for hypertension management in the rural African context along the continuum of care: screening, diagnosis, monitoring, treatment, follow up and BP control. Although based on indicators developed in North America and Europe, the ones presented here are modified for low resource settings. The indicators included: percentage of all visits with BP measurements; percentage of visits by hypertensive patients with hypertension diagnosis recorded in the chart; percentage of visits by hypertensive patients with physical exam or urinalysis; percentage of visits by hypertensive patients with at least one medication; number of total visits by hypertensive patients; and percentage of hypertensive patients with BP control. A target of 80% for process indicator percentages was used.

To view change over time, process indicators were calculated as proportions across clinics, plotted by time since intervention start. The time since intervention start is the difference between the date of the visit and the date the BHOMA intervention started at the clinic, in 6 month intervals. Although the main units of analysis were “visits” for process indicators (each 6 month interval only includes visits in that time period), we were also interested in examining patients (each 6 month interval includes all cumulative people up to that time period), which required classifying people as either hypertensive, or not. Because patients made different numbers of clinic visits (and thus have different numbers of BP recordings), all BP measurements were collapsed into a single median BP measurement per person to capture the average for classification purposes. Median was chosen over mean to minimize outlier impact from “white coat hypertension”. A person was classified as having hypertension if their median SBP ≥140 mmHg, or median DBP ≥90 mmHg, or if they were ever on antihypertensive medication. For the outcome indicator on BP control, in each 6 month interval the hypertension classification was recalculated to accommodate data from new visits. Only hypertensive patients with at least 2 visits were included, with categorization as “controlled” if their SBP <140 mmHg and DBP <90 mmHg at the most recent visit. All analyses were conducted in Stata/SE 13.1 (StataCorp, College Station, TX).

Based on these results, the qualitative in-depth interview guide was developed to detail possible mechanisms behind the quantitative data. Questions focused on the theoretical knowledge and actual practice surrounding screening, monitoring and treatment of hypertension at the clinics. In addition, quantitative results were presented to interviewees to elicit possible mechanisms behind the data. Thematic content analysis of the interview transcripts was conducted by two authors in iterative steps. After preliminary transcript review, a set of codes representing similar ideas or themes was developed. Using NVIVO 10, transcripts were coded by the two authors, with periodic comparisons and any coding conflicts resolved through discussion. During each round, any new concepts or emerging themes were created into new codes, and applied to all transcripts. Selected illustrative quotations are presented here.

## Results

From Jan 2011 to Dec 2014, a total of 1,021,530 total visits were made at the primary care clinics, with 318,380 visits (31.2%) made by adults ≥ 25 years. These visits, which include repeat visits, constituted our main quantitative analysis group. Of these, 62,543 visits (20.6%) with hypertension were recorded, made by 26,363 people.

Most health facilities had either a nurse or clinical officer serve as the clinic in-charge (16/20), with 3/20 staffed by an environmental health technician (a non-clinically trained cadre), and one clinic with only a security guard present on the interview day. All except 2 facilities had a working BP machine, with 10/20 digital and 13/20 analog. The average time to restock common medications in all clinics was less than 1 month.

### Hypertension screening and diagnosis

**Fig. 1:**
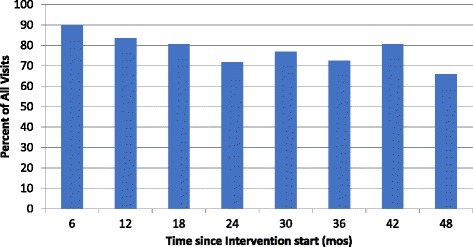
Screening process indicator: Percent of all visits with blood pressure measured, by time from intervention

When plotted by time since intervention start, the process indicator for screening (percent of total visits with BP measurement) ranged from 65 to 90%, with large variation between clinics (Fig. 1). Smaller clinics, and facilities located in Luangwa district, were more likely to consistently meet the target of 80%. Across time, all clinics had their highest percentages in the first year after intervention start.

**Fig. 2:**
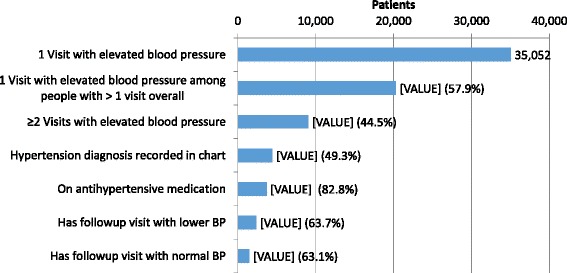
Cascade diagram of patients in rural primary care clinics, Zambia

**Fig. 3:**
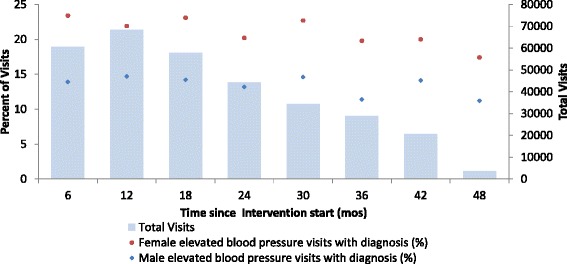
Diagnosis process indicator: Proportion of visits by hypertensive patients with recorded diagnosis, by time from intervention start

Figure 2 shows the cascade diagram of hypertensive patients from elevated BP measurement to normal BP at a follow up visit. The proportion of visits by hypertensive patients with recorded diagnosis was very low (19.1% overall). The proportion was lower for males than females (13.9% vs 21.9%, *p* < 0.0001), and did not appreciably increase with time (Fig. 3).

In the in-depth interviews, healthcare providers demonstrated strong knowledge of screening guidelines (BP taken at every visit as routine vital sign). In explaining the decreasing trend of visits with BP measurement, providers cited practical difficulties— principally equipment and personnel shortages:

*Some of the challenges include the BP machines, they are not properly functional and the other challenge at times is the personnel, in terms of us the trained personnel, we are usually overwhelmed. (provider at Chiawa clinic, in Kafue district)*

Analog and digital BP machines were used. Broken machines and batteries were difficult to replace, as clinic supply budgets were insufficient and erratic for needed resources. Indeed, 2/20 clinics did not have a functioning machine onsite during the time of the audit. Providers also stated the clinic support workers who usually take vital signs are insufficiently trained and are not able to use analog machines, which require use of stethoscopes. Insufficient and poorly motivated personnel constituted the other major challenge, described by half of clinics.

Reasons for low proportions of hypertension diagnoses were multifold. While most providers correctly identified a DBP ≥90 mmHg as hypertensive, there was more variation in the reported SBP threshold for hypertension classification (120–150 mmHg). Another important theme was the need for repeat visits of elevated BP before making a formal diagnosis of hypertension (range of 2–4 minimum visits). Lastly, there was a widespread belief among healthcare providers that stress was a common transient cause of elevated BP, and needed to be ruled out:

*Sometimes one can give a high reading due to circumstances, maybe stress, maybe some other happenings in day to day life…We can ask such a patient to be coming at regular intervals, maybe on daily basis for a week or so for us to say this is a confirmed case of hypertension. (provider at Chilanga clinic, in Kafue district)*

### Hypertension monitoring

To assess hypertension monitoring, Table 1 shows the proportion of visits by hypertensive patients with either a recorded physical examination or urinalysis. Physical examination included either a head and neck exam, a cardiovascular exam or a neurological exam. Both process indicators were consistently low at <10% across time.

**Table 1:** Monitoring process indicator: Proportion of visits by hypertensive patients with physical exam or urinalysis

Time after Intervention tart (mos)	Visits with physical exam	Visits with urinalysis checking protein
6	5.4% (727/13393)	0.16% (22/13393)
12	5.5% (754/13619)	0.17% (23/13619)
18	6.1% (688/11277)	0.07% (8/11277)
24	7.2% (591/8168)	0.04% (3/8168)
30	6.8% (453/6665)	0.03% (2/6665)
36	5.4% (278/5140)	0.08% (4/5140)
42	5.2% (191/3668)	0.05% (2/3668)
48	5.1% (31/613)	0% (0/613)
Total	6.0% (3682/61930)	0.1% (64/61930)

Healthcare providers demonstrated limited knowledge about the complications and comorbidities associated with hypertension. The most commonly mentioned complication was retinopathy, at 7 out of 20 providers. In practice, diabetes mellitus and renal failure are almost never evaluated at the primary care level due to lack of supplies:

*Because urinalysis testing was one of the routine procedures in any given institution. But as time went by, those things were no longer available…So, now I don’t know whether the government is going poorer and poorer to buy these urinalysis bottles for us, or to buy these urine sticks for us. We don’t have. So us as health workers, we work according to what we have. What we don’t have, less work. (provider at Kasenga clinic, in Chongwe district)*

The interview with a representative of the central medical distribution agency in Zambia revealed a urinalysis stick shortage for the past 5 years. The limited amount of available supplies is largely reserved for pregnant women to check for preeclampsia, and for patients initiating antiretroviral therapy.

When presented with the physical exam process indicator, a third of health care providers were genuinely surprised. In trying to explain the data, providers suggested that the combination of too few properly trained staff combined with the time pressure of a busy clinic resulted in a tendency to focus solely on the patient’s presenting complaint:

*The number of people that you would be expecting, so you just go direct on the real complaint of the patient, not like maybe you start checking from head to toe and the like. So sometimes when you look at the queue, “When am I going to finish with these people”. Why am I examining the whole body when I know what the patient is presenting with. (provider at Kanakantapa clinic, in Chongwe district)*

### Hypertension treatment and control

Overall, 21.1% of visits by patients with hypertension had a prescribed antihypertensive medication. The proportion of visits by hypertensive patients on an antihypertensive drug, starts at 21.4% at 6 months after intervention start, and decreases slightly over time to 15.7% at 48 months (Additional file 1: Table S1).

The most commonly prescribed antihypertensive medications at visits by people with hypertension in the clinics were nifedipine (10.5%) and furosemide (8.7%). Table 2 illustrates the antihypertensive drug stocks found at the 20 clinics surveyed in the facility audit, which differs from WHO recommended therapy for hypertension in developing countries [19]. Nifedipine (17/20), furosemide (15/20), hydrochlorothiazide-amiloride (known as Modiuretic) (9/20), and atenolol (9/20) were the most commonly available drugs. Furosemide had the highest availability at an average of 524 pills per clinic. Stockouts in the last 6 months were common.

**Table 2:** Antihypertensive drug and supply stocks related to hypertension management in rural primary care clinics, Zambia

	Clinics (*n* = 20)			
Drugs and Supplies	Usually Provides	Available today	Average available number of units per clinic^a^	Stockout in last 6 mos
	n (%)			
Atenelol	9 (45%)	3 (15%)	100	16 (80%)
Enalapril	0	0	0	20 (100%)
Furosemide	15 (75%)	10 (50%)	524	9 (45%)
Hydralazine	2 (10%)	2 (10%)	1	19 (95%)
Hydrochlorothiazide	0	0	0	20 (100%)
Hydrochlorothiazide-amiloride	9 (45%)	5 (25%)	226	15 (75%)
Methyldopa	7 (35%)	4 (20%)	100	14 (70%)
Nifedipine	17 (85%)	6 (30%)	35	15 (75%)
Propranolol	6 (30%)	2 (10%)	35	18 (90%)
Spironolactone	0	0	0	20 (100%)
glucometer strips	2 (10%)	0	0	20 (100%)
urinalysis sticks	11 (55%)	8 (40%)	35	13 (65%)

^a^ rounded to nearest whole number

**Fig. 4:**
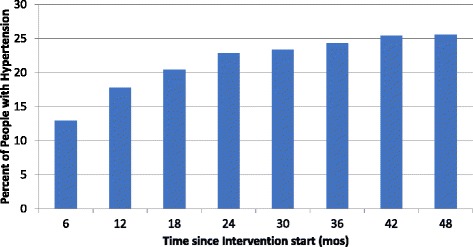
Blood pressure control outcome indicator: Percent of all hypertensive people with at least 2 visits who achieved BP < 140/90, by time from intervention

Among people with hypertension who made at least 2 visits, blood pressure control started at 12.9% at 6 months after intervention start, and gradually plateaued at around 25% (Fig. 4). Because each 6 month interval includes all cumulative people up to that time point, while the overall percent of controlled hypertensives increased, the rate of that change did not. In interviews, healthcare providers displayed varied knowledge levels about hypertension treatment. For initial treatment, half mentioned lifestyle changes (reduction in salt intake, exercise), while the other half started with drugs. First line medication preference was heterogeneous, with 10/20 listing furosemide, 10/20 nifedipine and 5/20 hydrochlorothiazide-amiloride. Recommended first line medication by the Zambian Ministry of Health is nifedipine or hydrochlorothiazide-amiloride [20]. The majority of providers opted to refer hypertensive patients who, after first line medication, were still uncontrolled at a follow up visit.

Stockouts and drug availability were reported to greatly influence antihypertensive drug prescription. When asked about why furosemide was so commonly used, despite not being a recommended first line drug, a provider explained:

*The reason is simple…We know in place of Lasix we should give Modiuretic, but we go ahead and give these Lasix even without slow K because that’s what we have at that particular time. We have ordered for Modiuretic several times, it has never come through. So we try, we outweigh, which one outweighs the other. If I was to give Lasix, for this person with 200+ BP, I give this person Lasix, or I let this patient go on—which one? (provider at Mphuka clinic, in Luangwa district)*

Furosemide is also provided in much larger containers (up to 1000 pills) than any other antihypertensives (up to 100). Inconsistent antihypertensive drug stock was mentioned by every provider, even though many are on the essential drug list and are in stock at the central distribution agency. The interview with the Medical Stores Limited representative revealed two potential reasons for this: 1) incorrect ordering of drugs by the clinic on their paper based forms, and 2) no transport to deliver ordered drugs from the District Medical Office to the clinic.

### Follow ups and provider preparedness

**Fig. 5:**
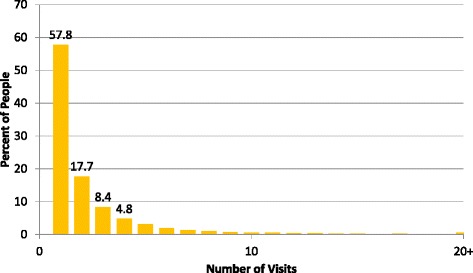
Number of visits by hypertensive patients presenting to rural primary care facilities, Zambia

A common challenge noted in all interviews was poor visit attendance. Over half of hypertensive patients presented only once to clinics during the 4-year study period (Fig. 5).

*Patients are being encouraged to be coming for checkups…But then sometimes because of distance, the patients won’t be coming for regular checkups. They prefer coming when they’re feeling bad, but when all is well, they wouldn’t come because they would look “Ah, just for a checkup, all that long, all that far.” (provider at Kanakantapa clinic, in Chongwe district)*

Providers believed that hypertension was not recognized or prioritized by the community, as a largely asymptomatic disease, with far distances contributing to the problem of nonattendance.

Another theme affecting care was self-reported provider preparedness with managing hypertension. Most healthcare providers stated they felt prepared to screen, manage and treat hypertension and other non-communicable diseases; however, half spontaneously listed furosemide as a first line drug.

## Discussion

Although the formal evaluation for BHOMA across disease entities is ongoing, this analysis of site data reveals important data about hypertension. Across these primary care clinics, despite high screening rates for hypertension, the process indicators for diagnosis, monitoring and treatment were poor and did not improve over time. The largest drop-offs in the cascade from elevated BP to controlled hypertensive appear to be lack of follow up visits and providers not formally diagnosing patients to initiate treatment. For BP control among hypertensive patients who made at least 2 visits (a small subset), while there was an increase in the overall cumulative percent, the rate of change did not increase. From qualitative interviews, challenges to hypertension management from the provider side included drug stock outs, lack of equipment, lack of knowledge despite reported self-confidence, and difficulty in getting patients to return for follow up visits. While BHOMA does incorporate process indicators into its multi-pronged intervention, the pre-identified set only included hypertension screening. The overall modest improvement suggests that despite initial strong efforts, the BHOMA intervention was not successful in overcoming these barriers for hypertension control.

A major limitation of this study is the lack of verified process and outcome indicators to use for evaluating hypertension care in developing countries, because most quality improvement indicators are created nationally by developed countries. While the screening, physical exam and diagnosis process indicators were compared to a target of 80%, the remaining indicators did not have an obvious standard (e.g., proportion of visits by hypertensive patients with urinalysis, or on treatment) [18]. These latter indicators are more dependent on clinical judgment and will require more extensive external review to determine appropriate targets. Other limitations include insufficient data on patient characteristics (ie smoking, educational level) to build logistic regression models to predict the likelihood of successful management. Patient compliance with visits or medications was also not assessed. For the qualitative data, social desirability bias may have been present in interviews. Furthermore, we only interviewed providers and did not seek patient perspectives on hypertension management.

Our findings are consistent with data from other developing countries. Given the BHOMA clinics represent almost all rural government facilities in Lusaka province, they are likely very representative of the Zambian population in particular, and perhaps rural southern African populations in general. The rapid drop-off from patients with elevated BP to formally diagnosed as hypertensive was consistent with data from the SAGE study in middle income countries, that showed among the hypertensive population, 66% were undiagnosed before the survey, with 73% untreated, and 90% uncontrolled [21]. As other surveys in low and middle income countries have shown, health care providers may not know the proper treatment for hypertension, and prescription patterns are seldom evidence based [22]. For example in our study, like in other low income contexts, thiazides were not commonly used despite being cheap and available [23]. In terms of sporadic care, as our data showed, patients may not think of hypertension as an important disease that needs regular follow up, with lack of symptoms as one of the most common reasons reported for not attending scheduled visits [24, 25]. Lastly, our finding that most providers in the clinics feel prepared to manage hypertension contrasted with the findings of the Zambia National Healthcare Strategic Plan, which states 50.7% of healthcare providers do not feel adequately prepared to manage hypertension in primary care settings [26]. Bias may have affected our interviews as admitting discomfort with hypertension management could be seen as tantamount to admitting incompetence.

Strong primary care systems have been shown to improve health [4]. In the US, geographical areas with higher primary care provider ratios to the population, but not higher specialist ratios, are associated with lower all-cause mortality and lower mortality from cardiovascular disease, cancer and stroke [27, 28]. However, there remain great difficulties in implementing evidence-based primary care interventions, with new research findings taking almost two decades to become widespread practice [29].

The disappointing data from the retrospectively generated process and outcome indicators presented here imply that a primary care quality improvement program will not necessarily improve performance indicator targets that are not explicitly pre-identified. In developed countries, performance indicators have been associated with greater likelihood of BP control in hypertensive patients if based on explicit evidence [11]. Furthermore, when coupled with financial incentives, they may drive positive health outcomes on a faster timeline, as in Rwanda where pay for performance since 2006 has increased institutional delivery, tetanus vaccine coverage and attendance at pediatric preventive care visits [5, 10, 30]. The data from this study is an important first step in developing Zambian appropriate targets for chronic non-communicable diseases. Additional file 2: Table S2 presents the structure, process and outcome indicators relevant for hypertension management, challenges and possible solutions.

Future research questions include patient and community member perspectives on non-communicable disease care, verifying quality improvement indicators and target standards that are relevant for hypertension in low resource settings, and evaluating pay for performance schemes to address challenges. The complete evaluation of BHOMA’s impact on community mortality and morbidity is ongoing with community surveys, and will elucidate if BHOMA has managed to improve community outcomes, even if the intermediate structure and process indicators for hypertension have not shown progress.

## Conclusions

In conclusion, this analysis presents data on process and outcome indicators for hypertension management in primary care clinics enrolled in the BHOMA study. While the screening process indicator was initially high, over time it decreased. Process indicators for diagnosis, monitoring and treatment were low overall and did not show improvement over time. The outcome indicator for blood pressure control did increase, but the rate of change did not. Challenges to hypertension management included supply stock outs, lack of knowledge and patient nonattendance at return visits.

## Additional files

Additional file 1: Table S1. Treatment process indicator: Proportion of visits by hypertensive patients with antihypertensive medication prescribed in rural primary care clinics, Zambia (DOCX 13 kb)
Additional file 2: Table S2. Challenges to hypertension management in rural primary care clinics, Zambia (DOCX 17 kb)

## Abbreviations

BHOMA: Better Health Outcomes through Mentoring and Assessment
BP: Blood pressure
CVD: Cardiovascular disease
DBP: Diastolic blood pressure
EMR: Electronic medical records
LMIC: Low- and middle-income countries
NCD: Non-communicable diseases
SBP: Systolic blood pressure
