# Supplementary material for: Hypertension management in rural primary care facilities in Zambia: a mixed methods study
Source: BMC Health Serv Res. 2017 Feb 3;17:111. doi: 10.1186/s12913-017-2063-0 (PMC5292001; doi:10.1186/s12913-017-2063-0)
Supplement: Additional file 1: Table S1. — Treatment process indicator: Proportion of visits by hypertensive patients with antihypertensive medication prescribed in rural primary care clinics, Zambia (DOCX 13 kb) [file 12913_2017_2063_MOESM1_ESM.docx]

**Additional file 1: Table S1. Treatment process indicator: Proportion of visits by hypertensive patients with antihypertensive medication prescribed in rural primary care clinics, Zambia**

| **Time since Intervention start (mos)** | **Visits with antihypertensive medication prescribed** |
| --- | --- |
| 6 | 21.4% (2862/13393) |
| 12 | 21.8% (2974/13619) |
| 18 | 21.3% (2399/11277) |
| 24 | 20.4% (1668/8168) |
| 30 | 22.6% (1503/6665) |
| 36 | 19.2% (987/5140) |
| 42 | 19.6% (718/3668) |
| 48 | 15.7% (96/613) |
| Total | 21.1% (13207/62543) |
