# Supplementary material for: Hypertension management in rural primary care facilities in Zambia: a mixed methods study
Source: BMC Health Serv Res. 2017 Feb 3;17:111. doi: 10.1186/s12913-017-2063-0 (PMC5292001; doi:10.1186/s12913-017-2063-0)
Supplement: Additional file 2: Table S2. — Challenges to hypertension management in rural primary care clinics, Zambia (DOCX 17 kb) [file 12913_2017_2063_MOESM2_ESM.docx]

**Additional file 2: Table S2. Challenges to hypertension management in rural primary care clinics, Zambia**

|  | **Challenges** | | **Proposed Solutions** |
| --- | --- | --- | --- |
| **Structure** How is care organized?  Stable elements that make up health system | •Equipment | Lack of analog/digital BP machines, digital BP machines run out of batteries, lack of urinalysis dipsticks and containers | Provide working analog/digital BP machines to each clinic, strengthen procurement chain for medical equipment (machines, batteries, urinalysis dipsticks and containers) |
|  | •Staff |  |  |
|  | ◦ Clinicians | Insufficient numbers and training of clinicians to screen, monitor, and treat hypertension | Increase number of trained clinicians, regular retraining of practicing clinicians |
|  | ◦ Support staff | Insufficient training of support workers to screen for hypertension with analog BP machines, volunteer attrition | Train support workers to use analog BP machines, task shift to support workers (vital signs, triage), regular retraining of new and practicing support workers |
|  | •Medications | Stockouts due to central stockouts, improper ordering by clinics, or lack of transport from district office | Regular retraining of clinicians and support workers on medication ordering in clinics, provide last mile transport from district office to clinics, strengthen procurement chain for medication |
| **Process** What is done? Interaction between patients and providers | •Screening |  |  |
|  | ◦Take BP of every patient at initial and follow up visits | Lack of equipment, lack of training in using analog BP machines (structural) | See above for structural solutions |
|  | ◦Diagnose patients having ≥2 visits with BP ≥140/90 mmHg with hypertension | Provider belief that >2 visits needed with elevated BP to make diagnosis, provider belief that much of elevated BP due to stress, patients nonattendance at follow up visits | Regular retraining of clinicians on hypertension diagnosis, community sensitization and incentivization on follow up visit attendance |
|  | •Monitoring |  |  |
|  | ◦Conduct physical exam, esp eye, cardiovascular, and neurological exam, at initial and follow up visits | Providers not valuing physical exam, provider belief in insufficient time to conduct thorough physical exam, poor provider motivation | Regular retraining of clinicians on importance of thorough physical exam, see above for structural solutions |
|  | ◦Conduct urinalysis to check for renal damage at initial visit | Lack of knowledge by providers, poor provider motivation, lack of urinalysis dipsticks and containers (structural) | Regular retraining of clinicians on proper use of urinalysis in hypertensive patients, see above for structural solutions |
|  | ◦Encourage patients to come to clinic for regular follow up | Distance to health facility, unrecognized importance of follow up in elevated BP | Community sensitization on complications of hypertension and other non-communicable diseases |
|  | •Treatment |  |  |
|  | ◦Encourage lifestyle changes in all patients with hypertension (reduce dietary salt and alcohol, stop smoking, lose weight, exercise) | Lack of knowledge by providers | Regular retraining of clinicians on encourgaing lifestyle changes in patients |
|  | ◦Start drug therapy if persistently elevated BP or if BP ≥160/100. | Lack of knowledge by providers, provider belief that much of elevated BP due to stress, patient nonattendance at follow up visits | Regular retraining of clinicians on drug therapy initiation and hypertension diagnosis, community sensitization and incentivization on follow up viist attendance |
|  | ◦Initial drug therapy should be a thiazide or calcium channel blocker | Lack of knowledge by providers, drug stockouts (structural) | Regular retraining of clinicians on drug therapy initiation, see above for structural solutions |
| **Outcomes** What happens to patients health?  End results of health care and nterventions | •Prevent new cases of hypertension | See Above | |
|  | •Control existing hypertension |  |  |
|  | •Reduce risk of developing long term consequences like cardiovascular disease, stroke, and renal disease |  |  |
|  | •Reduce mortality from cardiovascular disease, stroke, renal disease |  |  |
